# Reliability Analysis of CFRP-Strengthened RC Bridges Considering Size Effect of CFRP

**DOI:** 10.3390/ma12142247

**Published:** 2019-07-12

**Authors:** Hui-Bing Xie, Yuan-Feng Wang

**Affiliations:** School of Civil Engineering, Beijing Jiaotong University, Beijing 100044, China

**Keywords:** bridge engineering, reliability, CFRP, size effect, series-parallel model

## Abstract

A reliability analysis of an existing structure and a carbon fiber-reinforced plastic (CFRP)-strengthened structure is commonly used to evaluate the effectiveness of strengthening. It also provides the basis for the calibration of the partial factors involved in strengthening design codes. As the fundamental data, the statistical characteristics of the CFRP tensile strength affect the evaluation result. In general, the statistical characteristics of the CFRP strength were obtained from laboratory experiments with small-scale specimens, which resulted in errors caused by the size effect. In this study, a probabilistic series-parallel model is developed to describe the size effect of CFRP. The CFRP fabric is divided into a set of representative volume elements. By numerically simulating the CFRP strength, the relationship between the number of representative volume elements and the mean and coefficient of variation (COV) of the CFRP strength is analyzed. A chi-square test is carried out to determine the distribution type of the CFRP strength. An analytical expression of the mean, COV, and the cumulated density function of the CFRP strength are derived. Finally, an existing bridge, which has operated for 41 years, is selected for the case study; it is strengthened by using CFRP fabric. Reliability indexes of the existing and the strengthened bridges are calculated to analyze the size effect on the reliability of the strengthened structure.

## 1. Introduction

Externally-bonded carbon fiber-reinforced plastic (CFRP) is widely used globally for strengthening the existing concrete structures because of its effectiveness and convenience of construction [1,2]. In recent years, some design specifications related to structure strengthening by CFRP have been issued. A reliability analysis is necessary to calibrate the partial factors involved in the specifications. Uncertainties of the CFRP strength and the geometrical dimensions caused by material processing are widely realized. The inherent variability of the CFRP composite materials has attracted considerable research attention to the assessment of the CFRP performance and the effect of CFRP on the strengthened structures from a probabilistic perspective. Plevris et al. [3] conducted the pioneer study on the reliability of a CFRP-strengthened reinforced concrete (RC) beam in flexural. Further, some researchers [4,5,6] have focused on the quantification of epistemic uncertainty and aleatory uncertainty in CFRP strength. In the reliability analysis of strengthened structures, the statistical characteristics of CFRP tensile strength are generally obtained from laboratory experiments. However, most of the experiments conducted thus far used small-scale specimens, which have higher strength than the material regularly used in structural strengthening because of the size effect. This resulted in certain errors of the evaluated reliability index and the calibrated partial factors. Thus, the size effect of CFRP should be considered in the reliability analysis of strengthened structures [7].

Moreover, experimental investigations on the size effect on the pull-out of CFRP reinforcement from concrete, [8] on the debonding failure of CFRP-strengthened beams, [9] and on the shear strength of glass fiber-reinforced plastic-reinforced beams [10] have been conducted. However, uncertainties involved in the material processing were not evaluated in these studies. The increasing dimensions of the material lead to more internal defects, which results in a decrease in the material strength. In 1939, Weibull [11] first proposed a random strength model and stated that the defects in a material can be modeled using the weakest link integral in a solid mechanics setting. In the Weibull weakest-link theory, macro-fractures initiated from one representative volume element (RVE) would result in the failure of the entire structure, similar to the failure of one link in a chain. Bazant et al. [12,13,14,15] stated that the size dependence of the probability distribution of material strength can be described by a finite weakest-link model, where each link represents an RVE and the cumulative distribution function (CDF) of the RVE strength can be derived from an atomistic fracture model and a multiscale hierarchical model. Le [16] studied the size effect on the reliability indexes of quasi-brittle structures that fail at macro-crack initiation from one RVE. Lei and Su [17] resorted to the weakest-link formulation for the cumulative failure probability to characterize the size effect on the strength distribution of quasi-brittle materials. Batdorf [18] adopted the weakest link theory to determine the number of isolated fiber fractures, double fractures, and multiples of arbitrary order as a function of stress.

Based on the weakest-link model, Harlow and Phoenix [19,20] proposed a chain-of-bundles probability model to analyze the strength of fibrous materials. They reviewed the results for the chain-of-bundles model of the strength distribution of unidirectional fiber composites. Some exact results for small-scale composites and bounds for the asymptotic strength distribution of a composite in a simple case were obtained. On the basis of the chain-of-bundles model, Bergman [21] refined the bounds on the asymptotic strength distribution of a composite, which are generalized to a more general failure model. Ruggieri et al. [22] presented an analytical method for the assessment of the failure probability of brittle materials exhibiting progressive cracking prior to cleavage fracture. The limiting distribution for the fracture stress, which is identical to the first asymptotic distribution of the smallest values, was derived on the basis of the chain-of-bundles probability model. Moshtaghin et al. [23] presented a random field-based size effect model based on the weakest-link theory to predict the longitudinal tensile strength of clear timber.

A considerable amount of researches have been conducted on the size effect of fiber-reinforced plastic (FRP) and its influence on the probability of an FRP fracture [24,25,26,27,28,29,30]. However, most studies were conducted at the material level instead of structure level. In this study, we propose a probabilistic model to describe the size effect of CFRP strength. The CFRP fabric is modeled as a series–parallel system with many independent RVEs. The relations between the statistical characteristics of the CFRP strength and the size of the CFRP fabric are studied using a Monte-Carlo simulation. The analytical expressions of the mean, COV, and the cumulated density function of the CFRP strength are also derived. To analyze the effect of size on the reliability of the strengthened structures, an existing bridge built in 1978 is chosen as a case and strengthened by CFRP, which is designed on the basis of the Specifications for Strengthening Design of Highway Bridges [31]. The reliability indexes of the strengthened beam, considering size effect or not, are calculated and compared. A reliability evaluation of the CFRP-strengthened structures would be more rational when the proposed model was adopted; further, the calibrated partial factor would be more accurate.

## 2. Size Effect Model of CFRP

**Figure 1:**
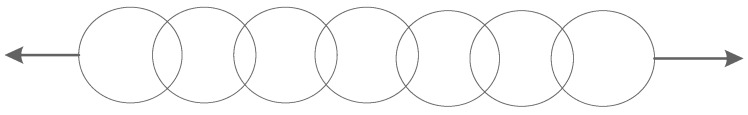
Illustration of the weakest link model.

In the weakest link model, the structure or material can be simplified as a one-dimensional chain, as shown in Figure 1. It is assumed that every link in the chain is independent and identically distributed. The failure probability of each link under stress *f* is identical and equals to *P_f,e_*(*f*). Thus, the failure probability of the chain under stress *f* could be expressed as Equation (1):

(1) $$P_{f,s}(f)=1-{\left[1-P_{f,e}(f)\right]}^{N}$$

where *N* is the number of links in the chain.

**Figure 2:**
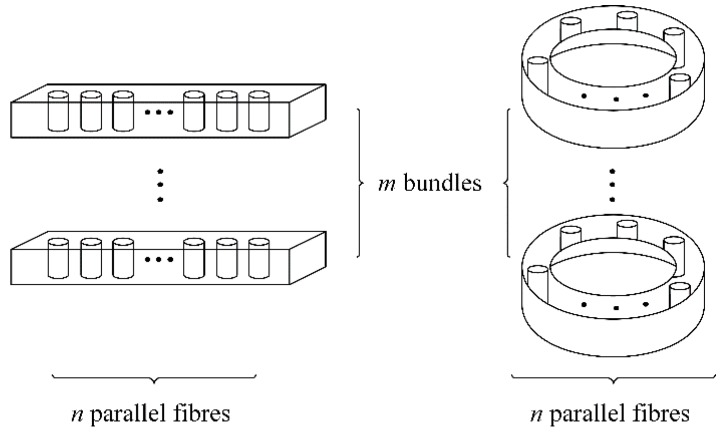
The composite is modeled as a chain with m links, each link consisting of a bundle with n parallel fibers [21].

For the chain-of-bundles model, the strength of a composite is modeled as the minimum strength of m bundles of fibers, each bundle containing *n* fibers, as shown in Figure 2.

**Figure 3:**
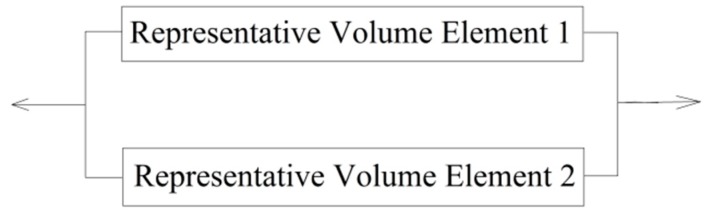
Sketch of a two-element parallel system.

To calculate the statistical characteristics of CFRP considering the size effect, a series-parallel model based on the weakest-link model and the chain-of-bundles model is proposed here. In the series-parallel system, which is commonly used for the modeling of bridge structures, all of the elements in the system were considered as plastic components; e.g., the elements could bear the load within the scope of their ultimate capacity as the load exceeds their strength. Taking a simple parallel system as an example (as shown in Figure 3), we assumed the elements to be plastic components and be mutually independent. The system failure probability could be expressed by the failure probability of the two elements, as shown in Equation (2).

(2) $$P_{f,s}=P_{f,e}^{2}$$

(3) $$P_{s,s}=1-P_{f,e}^{2}$$

where $P_{f,s}$ is the failure probability of the system, $P_{f,e}$ is the failure probability of the element, and $P_{s,s}$ is the system reliability.

However, CFRP is a kind of brittle material, which would rupture in the case of a failure, and thus, the stress in the system would be redistributed. The remaining elements would share the load that should have been carried by the failed one. This phenomenon would raise the failure probability of the remaining elements. The failure probability and the reliability of the two-element parallel system, as shown in Figure 3, can be calculated using Equations (4) and (5), respectively.

(4) $$P_{f,s}=P_{f,e}^{2}+C_{2}^{1}\cdot P_{f,e}\cdot P_{s,e}\cdot P_{f,e}^{\prime }$$

(5) $$P_{s,s}=P_{s,e}^{2}+C_{2}^{1}\cdot P_{f,e}\cdot P_{s,e}\cdot P_{s,e}^{\prime }$$

where $P_{f,e}^{\prime }$ is the failure probability of the components after stress redistribution; and $P_{s,e}^{\prime }$ is the reliability of the components after stress redistribution, $P_{s,e}^{\prime }<P_{s,e}$.

Comparison of Equations (2) and (4) revealed that the plastic system is considerably safer than the brittle system. Thus, CFRP should be modeled using a brittle system to avoid overestimation. In the proposed model for the size effect simulation of CFRP, the selection of a rational RVE and load sharing rule was very important. In this study, 15-mm-wide and 130-mm-long CFRP fabric, which was commonly used in the previous experimental studies [32], was chosen to be the RVE. The mean and COV of the strength of the RVE were derived from other experimental studies [33], as listed in Table 1.

After the fracture of the RVE, the total load was shared by the unfractured RVEs according to a load-sharing rule. In the past decades, some studies were conducted on the improvement and application of load sharing rules for fiber composite with several elements failed. There are three most commonly used rules: (a) Equal Load Sharing (ELS) [34] in which the distributed load is equally shared to the other cells within the material or bundle; (b) Local Load Sharing (LLS) [35] where the transferred load is only shared with the nearest neighbors; and (c) Hierarchical Load Sharing (HLS) [36,37] where the load of the failed elements are redistributed via a hierarchical structure to a neighborhood whose size is of the same order as the size of the failed region. For the tensile strength test on FRP fabric, stress concentration would occur after one fiber fractures. LLS or HLS methods are more suitable for modeling the stress redistribution in the CFRP. For the FRP fabric discussed in this study, it is bonded on the concrete beam; stress in the RVE depends on the tensile strain of the concrete surface. All the parallel RVEs could be regarded as independent of each other. After the fracture of an RVE, the stress in the RVE would release, leading to larger deformation of the concrete beam. Strain in the subface of concrete beam would increase uniformly. Correspondingly, the stress transferred to the other parallel RVEs would also increase uniformly. It could be regarded that stress in the fractured RVE redistributes on the other parallel RVEs equally. Thus, in this study, the ELS theory is selected to be the load-sharing rule to model the CFRP strips considering the size effect. 

**Figure 4:**
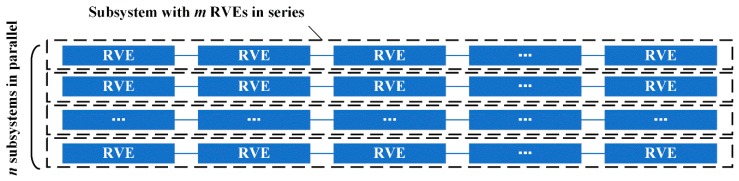
Series-parallel model of the CFRP used in the strengthen scheme.

To calculate the statistical characteristic of CFRP tensile strength, a numerical simulation method was conducted. Firstly, numbers of virtual CFRP specimens were established and modelled by the proposed series-parallel system. Each specimen contains *m* × *n* RVEs, as shown in Figure 4.

The strength of each RVE was generated through random sampling based on the statistical characteristics in Table 1.

Secondly, a series of predetermined loads were applied on the virtual specimens. The predetermined loads vector is listed in Equation (6):

(6) $$F_{i}=\left[n\cdot (f+(i-11)\sigma )\right],\begin{array}{ccc} & i=1,2,3\cdots 21 & \end{array}$$

where *f* and σ are the mean and standard deviation of CFRP RVEs’ tensile strength, which has been given in Table 1.

Failure probability of CFRP under different loads is calculated by Equation (7):

(7) $$P_{f,i}=\frac{x_{i}}{N}$$

where *x_i_* is the number of failed specimens under load $F_{i}$, and *N* is the total number of virtual specimens.

Finally, the mean and standard deviation of the CFRP specimens’ tensile strength could be obtained by Equations (8) and (9).

(8) $$\mu_{CFRP}=\sum_{i=1}^{21}F_{i}\cdot \left(P_{f,i}-P_{f,i-1}\right)$$

(9) $$\sigma_{CFRP}=\sqrt{\sum_{i=1}^{21}{\left(F_{i}-\mu_{CFRP}\right)}^{2}\cdot \left(P_{f,i}-P_{f,i-1}\right)}$$

Distribution type of the CFRP specimens’ tensile strength could be determined by $\chi^{2}$ test.

**Figure 5:**
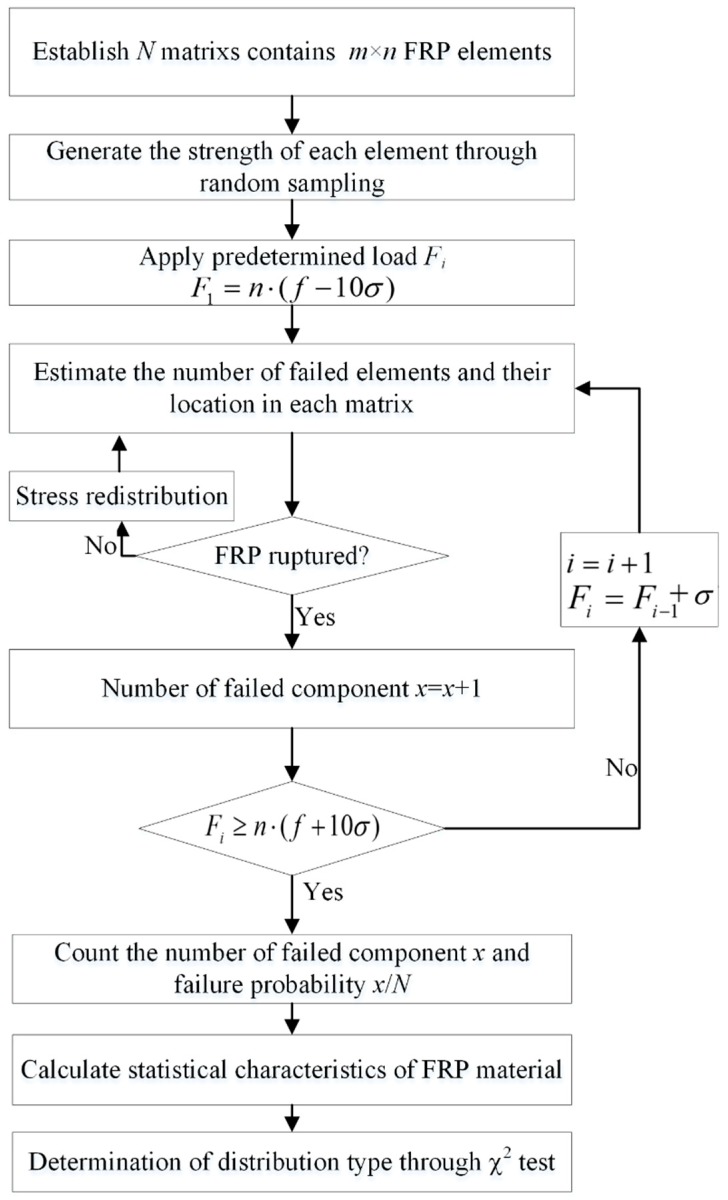
Flow chart of the numerical simulation process for determining the statistical characteristics of the CFRP strength considering the size effect.

A flow chart of the numerical simulation process is presented in Figure 5.

### 2.1. Mean CFRP Strength

**Figure 6:**
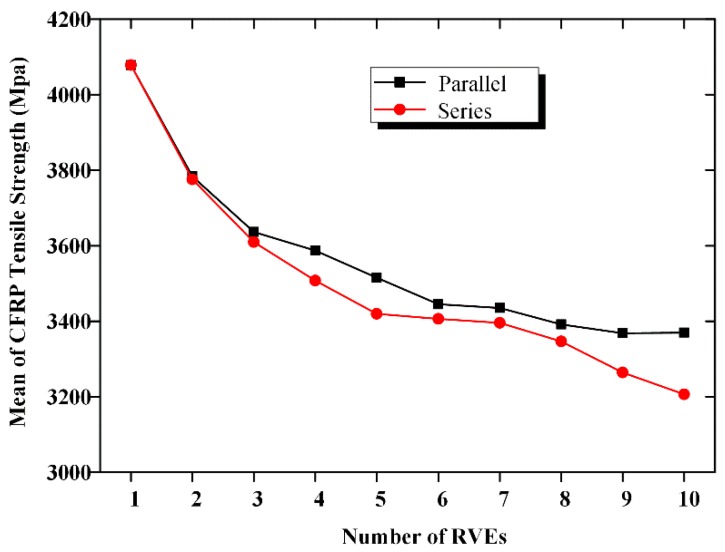
Mean strength of CFRP.

**Figure 7:**
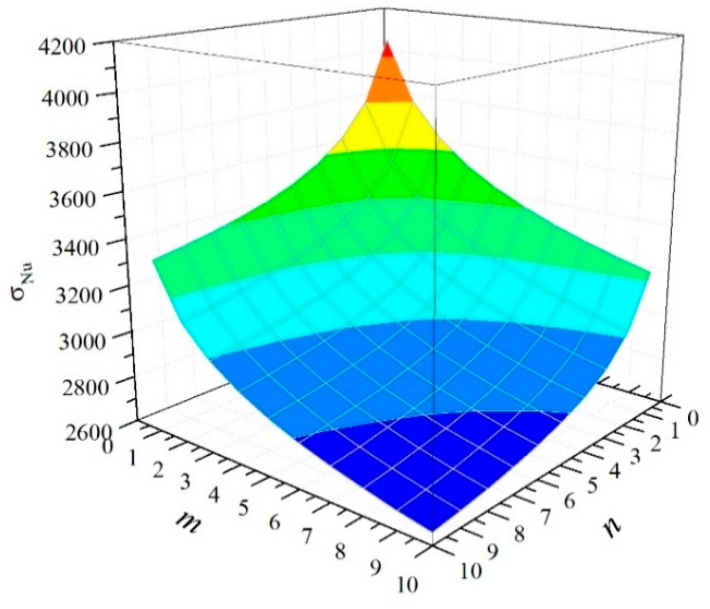
Relationship between mean strength and system size.

A numerical simulation method is used in this study to analyze the strength of CFRP fabric as a *m* × *n* series–parallel system, according to the failure criterion described above. Figure 6 shows the mean of the CFRP tensile strength along with an increase in *m* and *n*. Figure 7 displays the curved surface to represent the relationship between the mean CFRP tensile strength and the dimensions of the CFRP material. We observe from the figures that the mean CFRP strength decreased with an increase in the number of elements. For CFRP specimen with 10 serial or parallel RVEs, the mean values of the strength were 21% and 16% lower than those of the RVE. For the 10 × 10 series–parallel system, the reduction of strength due to the size effect is close to 40%.

### 2.2. COV of CFRP Strength

**Figure 8:**
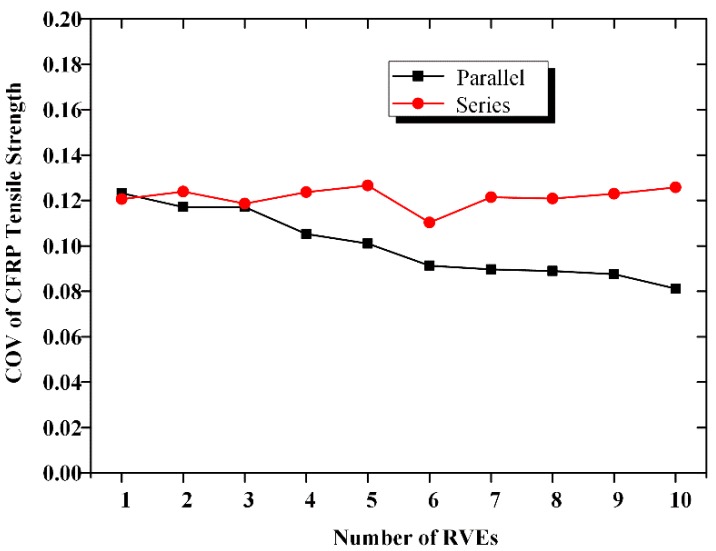
COV of CFRP strength.

**Figure 9:**
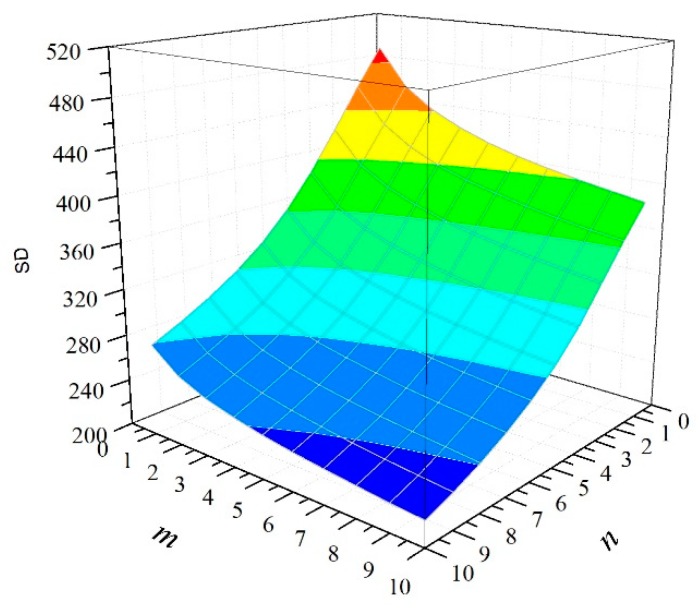
Relationship between standard deviation of strength and system size.

Figure 8 shows the relationship between COV of the CFRP tensile strength and the number of RVEs. With an increase in *n*, for the *n*-element parallel system, the COV of the CFRP strength decreases slightly. In contrast, for the *m*-element series system, COV remains almost the same with an increase in *n*. We thus conclude that the uncertainties related to CFRP decrease with an increase in the fabric width. Figure 9 presents the relationship between the standard deviation (SD) of the CFRP strength and the size of the CFRP material. We observe from the curve surface that the SD of the CFRP strength decreases with both the number of series elements and the number of parallel elements.

### 2.3. Distribution Type of CFRP Strength

To determine the distribution type of the CFRP strength, a chi-square test was conducted using the data obtained through the numerical simulation. Four types of commonly used distributions, namely normal distribution, log-normal distribution, Weibull’s distribution, and gamma distribution, are considered in the test. The results of the chi-square test are presented in Table 2. In the table, the first two columns present the number of RVEs. The third column shows the threshold value of the chi-square test with an assurance rate of 99.5%. In the last four columns, the test results are presented and the minimum values are marked in boldface. These results showed that the CFRP strength obeyed the Weibull distribution, which is in accordance with the experimental results obtained by Zhou et al. [7].

**Figure 10:**
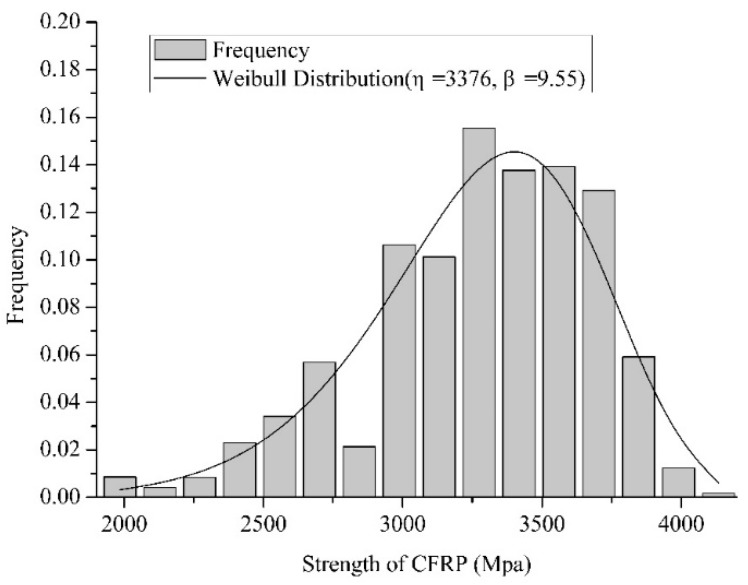
Comparison of the histogram and PDF of the 10-element series system.

**Figure 11:**
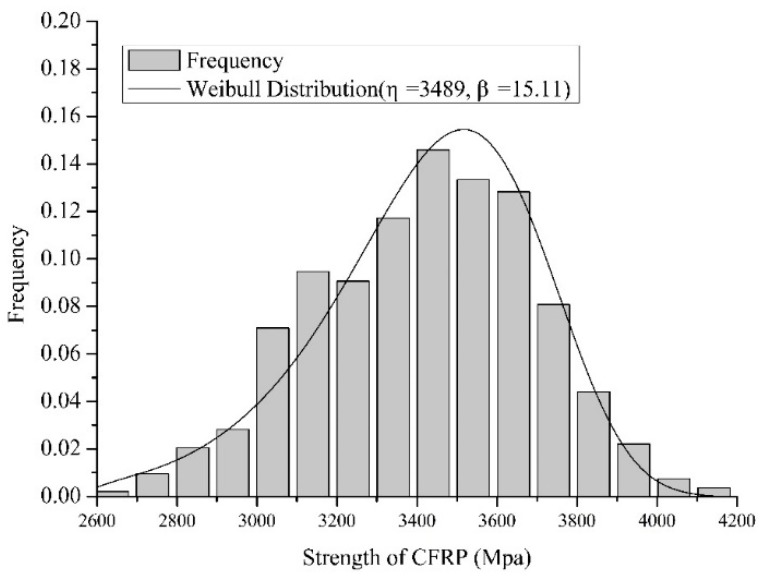
Comparison of the histogram and PDF of the 10-element parallel system.

Figure 10 and Figure 11 show a comparison of the histogram and the probability density function (PDF) of the 10-element series system and the 10-element parallel system, respectively.

For the convenience of calculation, the analytical expressions of the mean, COV, and the cumulated density function of the CFRP strength are derived; they are expressed as Equations (10)–(12), respectively.

(10) $$\mu_{s}=f_{FRP}\cdot \frac{\eta }{n{\left(-m\right)}^{1/\beta }}\sum_{i=1}^{n}\left[\ln(1-\frac{i}{n+1}\right){]}^{1/\beta }$$

(11) $$\sigma_{s}=\sqrt{\frac{1}{n}\sum_{i=1}^{n}\left[1.63\cdot \ln(1-\frac{i}{n+1}\right){]}^{2/\beta }-1}$$

(12) $$F(x,m)=\sum_{i=1}^{m}C_{m}^{i}\frac{{\left[e^{{\left(x/\eta \right)}^{\beta }}-1\right]}^{i}}{e^{m\cdot {\left(x/\eta \right)}^{\beta }}}\cdot F(\frac{mx}{m-i},m-i)$$

where *η* is the scale parameter, *β* is the shape parameter, *n* is the number of the parallel elements, *m* is the number of series elements, and *f*_FRP_ is the mean strength of the RVEs.

**Figure 12:**
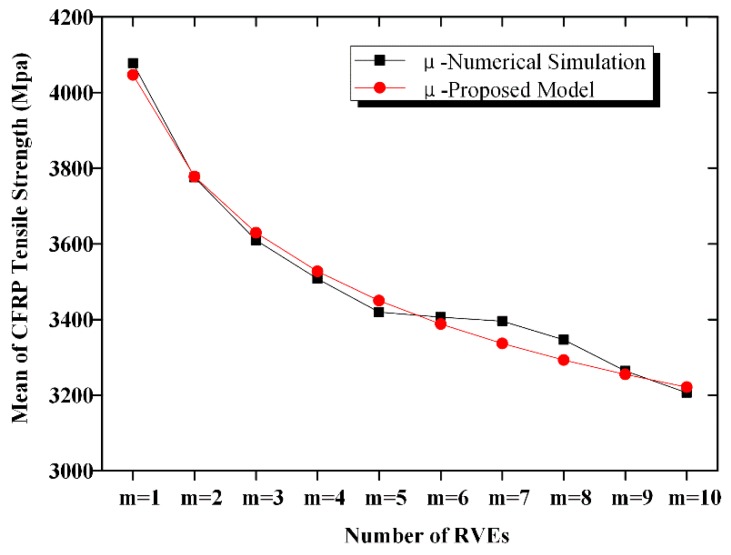
Comparison of the mean strength of the n-element series system.

**Figure 13:**
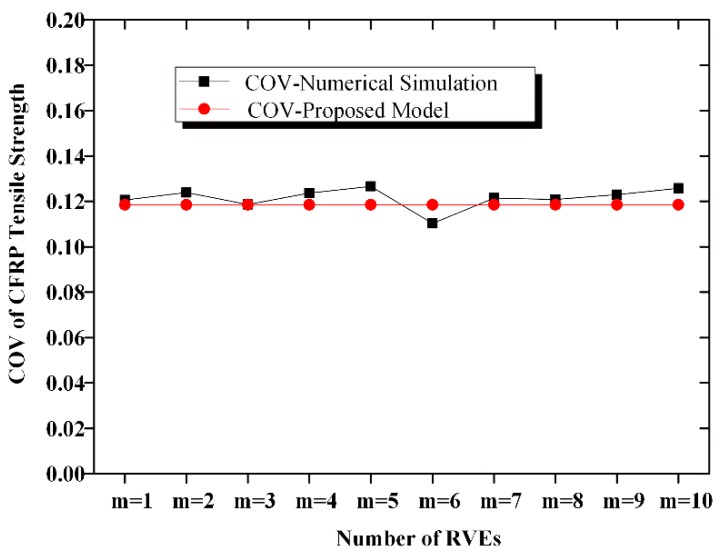
Comparison of COV of the CFRP strength of the n-element series system.

**Figure 14:**
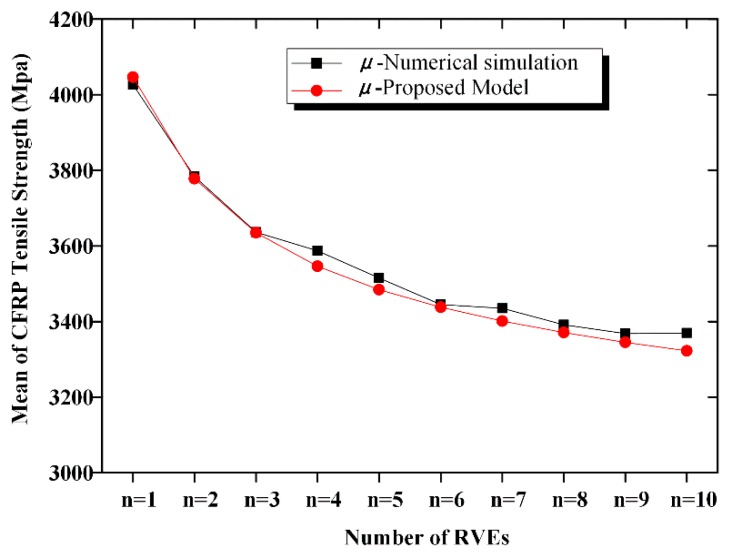
Comparison of the mean strength of the n-element parallel system.

**Figure 15:**
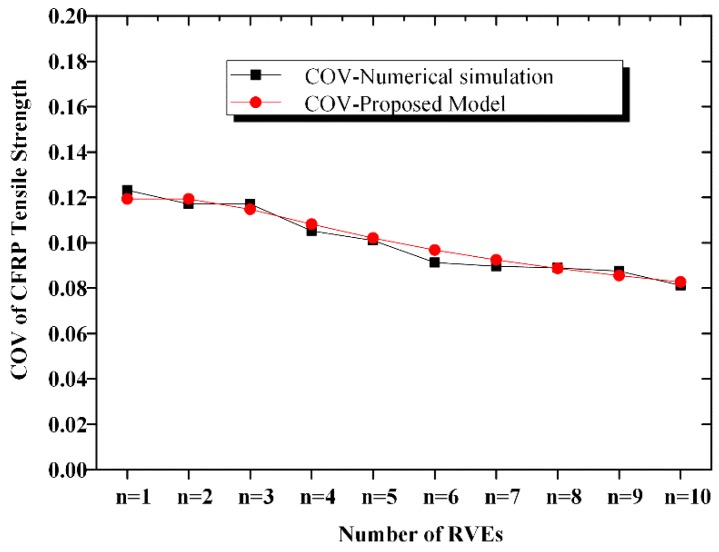
Comparison of COV of the CFRP strength of the n-element parallel system.

A comparison of the calculated mean and COV according to Equations (10) and (11) with the simulated mean and COV is shown in Figure 12, Figure 13, Figure 14 and Figure 15. The calculated results agreed well with the simulated ones; hence, the proposed equation could be used to calculate the strength and COV of the CFRP strength.

## 3. Case Study

To analyze the size effect on the reliability of a CFRP-strengthened structure, a five-span simply-supported bridge with RC beams constructed in 1978 is selected for the case study. The selected bridge is strengthened by externally epoxy-bonded CFRP. The strengthening scheme is designed on the basis of the *Specifications for Strengthening Design of Highway Bridges* [31], and the reliability index of the strengthened bridge is calculated to analyze the influence of size effect on the reliability.

### 3.1. Project Overview

**Figure 16:**
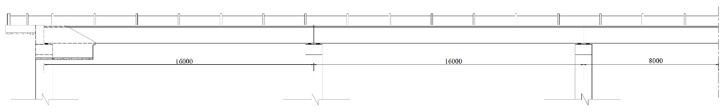
Side elevation of the bridge (mm).

**Figure 17:**
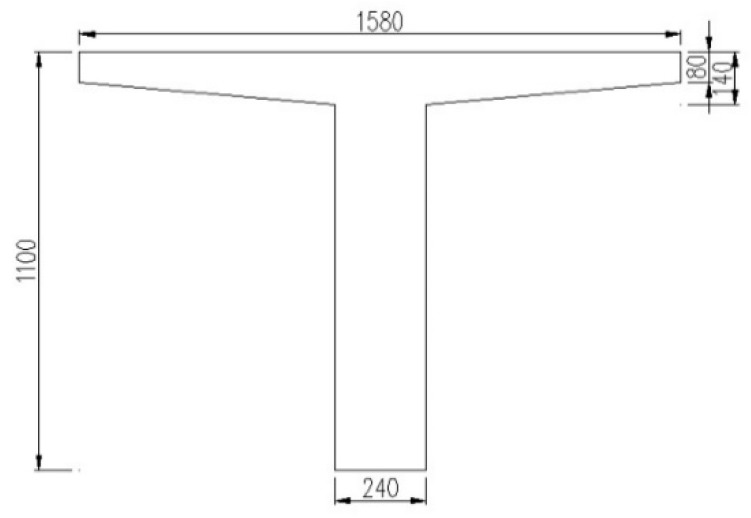
Cross section of the internal beam (mm).

The span length of the case bridge is 16 m, and the total length of the bridge is 80.62 m. The width of the bridge is 13.5 m and eight reinforced concrete T beams are arranged paralleled in a single span. The nominal strength of the concrete used in the bridge is 30 MPa. The area of tensile rebar reinforcement is 4926 mm^2^, and the nominal strength of the rebar is 345 MPa. The design load was a superposition of the traditional HS20 truck and lane loading. The side elevation of the bridge and the cross section of the internal beam are shown in Figure 16 and Figure 17.

In 2011, a regular inspection was conducted on the considered bridge. The condition of the bridge was classified as Condition State IV, implying that rehabilitation was needed according to *Code for Maintenance of Highway Bridges and Culvers* [38]. In this study, the strengthening of the existing bridge is designed on the basis of the *Specifications for Strengthening Design of Highway Bridges*. The resistance of the strengthened girders is calculated using a sectional analysis. There are five common failure modes for the FRP-strengthened concrete beam [38,39].

(1) Crushing of the concrete in compression before yielding of the reinforcing steel;
(2) Yielding of the steel in tension followed by rupture of the FRP laminate;
(3) Yielding of the steel in tension followed by concrete crushing;
(4) Shear/tension delamination of the concrete cover;
(5) Debonding of the FRP from the concrete substrate.

In this study, failure modes 1, 4 and 5 are deemed to be avoided through rational design and anchorage measures, such as U-wrap and development length. Failure modes 2 and 3 are considered in this study:

1. Steel-yielding concrete crushing. In this mode, the tensile strain of the CFRP laminate is less than the allowable tensile strain when a failure occurrs. The flexural capacity of the strengthened RC beam can be expressed by equilibrium of the moment at the mid-span cross-section, as Equation (13) [31]:

(13) $$R=f_{c}bx(h_{0}-\frac{1}{2}x)+f_{s}(1-\alpha )A_{s}(h_{0}-a_{s})+E_{f}\varepsilon_{f}A_{f}a_{s}$$

where *f_c_* is the 28-day yield strength of concrete; *b* is the width of the girder web; *x* is the height of the compression zone, which can be calculated by equilibrium of forces at the mid-span cross-section, as Equation (14); *h_0_* is the effective height of the girder cross section; $f_{s}^{\prime }$ is the yield strength of the compression reinforcement; *α* is the corrosion rate of the reinforcement; $A_{s}^{\prime }$ is the area of compression reinforcement; *a_s_* is the thickness of the concrete cover; *E_f_* is the elastic module of the CFRP; $\varepsilon_{f}$ is the CFRP strain when the failure occurred; and *A_f_* is the sectional area of the CFRP.

(14) $$f_{c}bx+f_{s}^{\prime }A_{s}^{\prime }(1-\alpha )=f_{s}A_{s}(1-\alpha )+E_{f}\varepsilon_{f}A_{f}$$

in which $f_{s}$ and $A_{s}$ are yield strength and area of tension reinforcement, respectively.

2. Steel-yielding FRP rupture. In this mode, the concrete has not yet reached its ultimate capacity when the failure occurrs. This is the most common failure mode of a well-designed CFRP-strengthened RC beam. The flexural capacity of the strengthened RC beam can be expressed as Equation (15):

(15) $$R=f_{s}(1-\alpha )A_{s}(h_{0}-a_{s}^{\prime })+E_{f}[\varepsilon_{f}]A_{f}(h-a_{s}^{\prime })-\int_{0}^{c}b\sigma (x)xdx+\int_{0}^{c}b\sigma (x)dxa_{s}^{\prime }\;\sigma (x)=f_{c}\left[2\left(\frac{\varepsilon (x)}{\varepsilon_{0}}\right)-{\left(\frac{\varepsilon (x)}{\varepsilon_{0}}\right)}^{2}\right]\;\varepsilon (x)=(1-\frac{x}{c})\varepsilon$$

where $A_{s}$ is the sectional area of the tension reinforcement; $\left[\varepsilon_{f}\right]$ is the ultimate strain in the FRP; σ(x) and ε(x) are the stress distribution function and strain distribution function in compressive concrete, respectively; $\varepsilon_{0}$ is the strain corresponding to the ultimate strength of concrete; ε is the strain of concrete while failure occurs; and c is the depth to the neutral axis, which can be calculated by Equation (16);

(16) $$\frac{\varepsilon }{\varepsilon +[\varepsilon_{f}]+\varepsilon_{1}}=\frac{c}{h}$$

where $\varepsilon_{1}$ is the initial strain in the concrete before being strengthened, which can be calculated by Equation (17). It is introduced to consider the discrepancy between the strain in CFRP and that in concrete surface.

(17) $$\varepsilon_{1}=\frac{M_{d1}x_{1}}{E_{c}I_{cr}}$$

where $M_{d1}$ is the initial moment; $x_{1}$ is the relative height of the compression zone of the cracked cross section; $E_{c}$ is elastic modulus of concrete; and $I_{cr}$ is the inertia moment of the cracked cross section.

**Figure 18:**
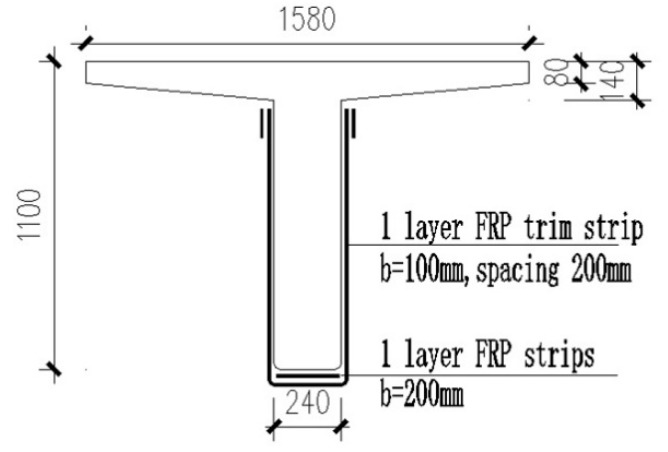
Cross section of the strengthened RC beam (Reinforcement scheme I).

The strengthening scheme is designed according to the Specifications for Strengthening Design of Highway Bridges [24]. In the Reinforcement scheme, a 200-mm-wide CFRP strip was epoxy-bonded to the soffit of the RC beam to enhance the flexural bearing capacity. The construction drawings of the strengthen scheme are shown in Figure 18.

### 3.2. Performance Function

The performance function for the flexural limit of a CFRP-strengthened RC simple-supported T beam is shown in Equation (18):

(18) $$Z=\gamma_{mfc}\cdot R-S=R-M_{D}-M_{L}$$

where $\gamma_{mfc}$ is the model uncertainty factor for the flexural capacity of CFRP-strengthened RC beams, $M_{D}$ is the bending moment generated by permanent loads, and $M_{L}$ is the bending moment generated by live loads.

The moment generated by permanent loads $M_{D}$ is expressed as Equation (19):

(19) $$M_{D}=\frac{l^{2}}{8}[(g_{1}+g_{2})\lambda_{conc}+g_{3}\lambda_{asph}]$$

where *l* is the span length of the RC girder, *g*_1_ is the weight of the precast RC girder per unit length, *g*_2_ is the equivalent dead load of the RC diaphragm per unit length, $g_{3}$ is the weight of the asphalt concrete pavement per unit length, *λ_conc_* is the uncertainty factor of the RC dead load, and *λ_asph_* is the uncertainty factor of the asphalt concrete dead load.

The moment generated by the live loads *M_L_* could be expressed as Equation (20):

(20) $$M_{L}=[I_{beam}\xi (1+c)(m_{cq}q_{k}\frac{l^{2}}{8}+m_{cq}P_{k}\frac{l}{4})+m_{cr}q_{r}\frac{l^{2}}{8}]\lambda_{mtrk-i}$$

where *I_beam_* is the impact coefficient of the vehicle load, *ξ* is the girder distribution factor, *c* is the overload rate, *m_cq_* is the transverse distribution coefficient of the vehicle load, $q_{k}$ is the uniform lane load, *P_k_* is the concentrated lane load, *m_cr_* is the transverse distribution coefficient of the crowd load, $q_{r}$ is the crowd load, and *λ_mtrk-i_* is the uncertainty factor of the moment generated by the live loads on the beam.

The statistical characteristics of the CFRP strength can be calculated using the above-proposed model. In the strengthening scheme, the width of the CFRP was 200 mm, and the length of the CFRP was 9.6 m, which contains an effective length of CFRP for flexure strengthening and development length. To avoid debonding of CFRP, U-wraps were applied as mechanical anchoring with an interval of 200 mm. The CFRP strip adhered on the RC beam could be modeled by a 74 × 13 series-parallel system. The statistical properties of the variables considered in this study are listed in Table 3.

### 3.3. Reliability Evaluation of the CFRP-Strengthened Bridge

A hybrid procedure is used for the estimation of the reliability index, in which descriptive statistics of performance function Z are obtained by using Monte-Carlo (MC) simulation, and the reliability of the existing bridge is assessed using the first-order reliability method (FORM), as shown in Equation (21).

(21) $$\beta =\mu_{z}/\sigma_{z}$$

Using this method, we calculated the reliability of the strengthened bridge, as listed in Table 4.

After 40 years of operation, the considered bridge has a reliability index lower than the target reliability specified in the *Unified Standard for the Reliability Design of Highway Engineering Structures* [40]. Table 4 shows that the strengthening of the RC bridge with CFRP improved the safety of the structure effectively, irrespective of the size effect on the CFRP strength. Moreover, we conclude that when the size effect of CFRP is not considered, the reliability of the strengthened structure would be overestimated. Although the reliability index considering the size effect is only 0.2 less than that without the consideration, a tenfold difference is observed between the corresponding failure probabilities.

## 4. Conclusions

The uncertainties involved in the CFRP have significant effects on the reliability of strengthened structures, and thus, the size effect of the CFRP strength should be considered in an analysis of the variability of CFRP.

A series–parallel model is proposed to describe the statistical characteristics of CFRP strength. With an increase in the fabric dimensions, the probability that a defect exists increases. Consequently, the mean CFRP strength decreases. For the 10-element series and the 10-element parallel systems, compared with the strength of the RVE, the mean strength decreases by 21% and 16%, respectively. However, the COV of the strength remains almost constant with an increase in the number of RVEs. A chi-square test is implemented; and it reveals that the strength of the CFRP obeys the Weibull distribution.

For the ease of calculation, we derive the analytical expressions of the mean, COV, and the cumulated density function of the CFRP strength. The computed results according to the derived model agree well with the numerical simulation ones.

To analyze the influence of size on the reliability evaluation of CFRP-strengthened structures, a five-span bridge constructed in 1978 is considered in the case study. In the reliability evaluation of the case bridge, two failure modes are considered. FRP debonding is deemed to be avoided by anchoring methods. This might result in overestimation of the reliability index. In this study, a comparison between the reliability indexes of the existing bridge and the strengthened bridge considering the size effect are conducted. It can be concluded from the result that CFRP strengthening of the RC bridge improves the safety of the structure effectively, irrespective of the size effect of the CFRP strength. With respect to the size effect of CFRP, the reliability of the strengthened structure is overestimated.

## Figures and Tables

**Table 1:** Statistical characteristics of CFRP [33].

Statistical Characteristics	Mean (MPa)	COV	Distribution Type
Value	4078	0.12	Weibull’s distribution

**Table 2:** Chi-square test results.

m	n	$\chi_{0.995}^{2}$ (16-2-1)	Normal Distribution	Log-Normal Distribution	Weibull’s Distribution	Gamma Distribution
1	1	29.819	3.450	8.599	**3.368**	4.400
2	1	29.819	9.200	15.326	**3.766**	11.615
3	1	29.819	6.097	9.519	**4.081**	8.396
4	1	29.819	9.892	13.070	**7.251**	11.684
5	1	29.819	11.219	14.616	**7.295**	12.863
6	1	29.819	9.236	12.792	**5.531**	11.287
7	1	29.819	**8.987**	9.544	12.003	9.368
8	1	29.819	14.790	**14.281**	24.031	14.394
9	1	29.819	14.349	**12.158**	33.040	12.845
10	1	29.819	**6.466**	7.619	6.974	6.867
1	2	29.819	**4.027**	6.265	4.708	5.273
1	3	29.819	7.315	12.884	**2.717**	9.794
1	4	29.819	12.472	16.008	**9.961**	14.372
1	5	29.819	10.716	16.062	**5.845**	13.210
1	6	29.819	15.178	25.477	**6.651**	23.487
1	7	29.819	2.854	**1.651**	16.599	1.898
1	8	29.819	27.733	33.521	**24.190**	30.065
1	9	29.819	11.805	19.860	**6.321**	14.722
1	10	29.819	16.747	21.015	**13.123**	18.953

**Table 3:** Statistical properties of variables [40].

Variables	Bias	COV	Distribution Type
Model uncertainty factor of CFRP-strengthened RC beam in flexure *γ_mfc_*	1.0980	0.1190	Normal
Yield stress of steel reinforcing *f_y_*	1.0900	0.0606	Normal
Reinforced steel area in concrete *A_s_*	1.0000	0.0350	Normal
28-day yield strength of concrete *f*_c_	1.2510	0.1464	Normal
Uncertainty factor: weight of concrete *λ_conc_*	0.9865	0.0980	Normal
Uncertainty factor: weight of asphalt *λ_asph_*	0.9891	0.1114	Normal
Impact on girders *I_beam_*	1.0000	0.0500	Gumbel
Uncertainty factor: live load moment on girder $\lambda_{mtrk}$	1.3500	0.162	Gumbel
Width of cross section *b*	1.0013	0.0081	Normal
Height of cross section *h_0_*	1.0124	0.0229	Normal
Design distribution of lane load *q_k_*	0.7882	0.1082	Gumbel
Crowd load *q_r_*	0.5786	0.3911	Gumbel
Tensile strength of CFRP laminates *f_FRP_* (size effect not considered)	1.1900	0.1260	Weibull
Tensile strength of CFRP laminates *f_FRP_* (size effect considered)	0.7512	0.0805	Weibull
Thickness of CFRP strips *t*	0.8250	0.0400	Normal
Elasticity modulus of CFRP laminates *E_f_*	1.1800	0.1040	Normal

**Table 4:** Reliability indexes of the considered bridge strengthened with different schemes.

	Existing Bridge	Reference Scheme	Reinforcement Scheme Considering Size Effect
Reliability index	4.1	5.4	5.0
Failure probability	2.07 × 10^−5^	3.33 × 10^−8^	2.87 × 10^-7^

## Abbreviations

FRP	fiber-reinforced plastic
CFRP	carbon fiber-reinforced plastic
GFRP	glass fiber-reinforced plastic
MC	Monte-Carlo
COV	coefficient of variation
RC	Reinforced concrete
RVE	representative volume element
CDF	cumulative distribution function
ELS	Equal Load Sharing
SD	standard deviation
PDF	probability density function
FORM	first-order reliability method

## Symbols

*P_f,e_*(*f*)	is the failure probability of a link under stress f;
*P*_f,s_(*f*)	is the failure probability of the chain;
N	is the number of links in the chain;
f	is the mean deviation of CFRP RVEs’ tensile strength;
σ	is the standard deviation of CFRP RVEs’ tensile strength;
*x_i_*	is the number of failed specimens under load $F_{i}$;
*N*	is the total number of virtual specimens;
$P_{f,s}$	is the failure probability of the system;
*e*	is the failure probability of the element;
$P_{s,s}$	is the system reliability;
$P_{f,e}^{\prime }$	is the failure probability of the components after stress redistribution;
$P_{s,e}^{\prime }$	is the reliability of the components after stress redistribution;
*η*	is the scale parameter;
*β*	is the shape parameter;
*n*	is the number of the parallel elements;
*m*	is the number of series elements;
*f*_FRP_	is the mean strength of the RVEs;
*f_c_*	is the 28-day yield strength of concrete;
*b*	is the width of the girder web;
*x*	is the height of the compression zone;
*h_0_*	is the effective height of the girder cross section;
$f_{s}^{\prime }$	is the yield strength of compression reinforcement;
$f_{s}$	is the yield strength of tension reinforcement;
*α*	is the corrosion rate of the reinforcement;
*a_s_*	is the thickness of the concrete cover;
*E_f_*	is the elastic module of the CFRP laminate,
$\varepsilon_{f}$	is the CFRP strain when the failure occurred;
*A_f_*	is the sectional area of the CFRP laminate;
$A_{s}^{\prime }$	is the sectional area of compression reinforcement;
$A_{s}$	is the sectional area of tension reinforcement;
$\xi_{fb}$	is the relative height of the compression zone during a CFRP rupture;
$\left[\varepsilon_{f}\right]$	is the ultimate strain in the FRP;
$\gamma_{mfc}$	is the model uncertainty factor for the flexural capacity of CFRP-strengthened RC beams;
$M_{D}$	is the bending moment generated by permanent loads;
$M_{L}$	is the bending moment generated by live loads;
*l*	is the span length of the RC girder;
*g*_1_	is the weight of the precast RC girder per unit length;
*g*_2_	is the equivalent dead load of the RC diaphragm per unit length;
$g_{3}$	is the weight of the asphalt concrete pavement per unit length;
*λ_conc_*	is the uncertainty factor of the RC dead load;
*λ_asph_*	is the uncertainty factor of the asphalt concrete dead load;
*I_beam_*	is the impact coefficient of the vehicle load;
*ξ*	is the girder distribution factor, *c* is the overload rate;
*m_cq_*	is the transverse distribution coefficient of the vehicle load;
$q_{k}$	is the uniform lane load;
*P_k_*	is the concentrated lane load;
*m_cr_*	is the transverse distribution coefficient of the crowd load;
$q_{r}$	is the crowd load;
*λ_mtrk-i_*	is the uncertainty factor of the moment generated by the live loads on the beam.
σ(x)	is the stress distribution function in compressive concrete;
ε(x)	is the strain distribution function in compressive concrete;
$\varepsilon_{0}$	is the strain corresponding to ultimate strength of concrete;
ε	is the strain of concrete while failure occurs; c is the depth to the neutral axis;
$\varepsilon_{1}$	is the initial strain in the concrete before strengthened;
$M_{d1}$	is the initial moment;
$x_{1}$	is the relative height of the compression zone of the cracked cross section;
$E_{c}$	is elastic modulus of concrete;
$I_{cr}$	is the inertia moment of cracked cross section.
